# Effectiveness of infection control measures informed by a modified Blue-Carba test in reducing rectal carriage of carbapenemase-producing bacteria in general wards: a prospective interrupted time series study

**DOI:** 10.3389/fphar.2025.1584646

**Published:** 2025-09-04

**Authors:** Maximiliano Gabriel Castro, Fernanda Argarañá, Carla Bernasconi, Leticia Margenet, Ana Paula Amato, Joaquín Ignacio Coduri Anthonioz Blanc, Erwin Alexander Rottoli, Manuel Protto, Macarena Vicino, María José Sadonio, Federico Rafael Galluccio, Héctor Mario Musacchio, Fernando Pasterán, Sonia Alejandra Gómez

**Affiliations:** ^1^ Internal Medicine Department, JB Iturraspe Hospital, Santa Fe, Argentina; ^2^ Medical Sciences Faculty, National University of the Littoral, Santa Fe, Argentina; ^3^ Microbiology Laboratory, JB Iturraspe Hospital, Santa Fe, Argentina; ^4^ Biochemistry and Biological Sciences Faculty, National University of the Littoral, Santa Fe, Argentina; ^5^ Consejo Nacional de Investigaciones Científicas y Tecnológicas (CONICET), Santa Fe, Argentina; ^6^ Servicio Antimicrobianos, Instituto Nacional de Enfermedades Infecciosas (INEI) - ANLIS “Dr. Carlos G. Malbran”, Ciudad Autónoma de Buenos Aires, Argentina

**Keywords:** carbapenem-resistant *Enterobacterales*, infection control, microbial sensitivity tests, carbapenemase, rectal carriage, antimicrobial resistance, AMR, diagnostics

## Abstract

**Introduction:**

The spread of carbapenemase-producing bacteria (CPB) is exacerbated in hospital settings, making the surveillance of rectal carriage of CPB crucial to halt their spread. However, the processing time until detection with traditional methods and the cost of new techniques limit their implementation. We aimed to evaluate the effectiveness of infection prevention and control (IPC) measures guided by a novel algorithm (NA) for rectal swab processing, which incorporated a modified Blue-Carba test (mBCT), in reducing carbapenemase-producing bacteria (CPB) rectal carriage prevalence in general wards of a tertiary-care hospital from Argentina. Additionally, we assessed the impact of this algorithm on microbiological turnaround time (mTAT) and time to positive results (TPR).

**Materials and methods:**

An experimental and quasi-experimental designs were combined into a prospective interrupted time series study structured in three phases: P1 (February 2022-July 2022), P2 (August 2022-January 2023; intervention) and P3 (February 2023-July 2023). Briefly, the NA included as key steps a 6-hour pre-incubation at 37 °C in nutrient broth, followed by a 15-minute centrifugation at 3,200 rpm. The mBCT was set at pH 10.7 using 9 mg of imipenem in a final volume of 150 μL and was validated against conventional methods testing 1,120 samples. It was subsequently implemented to assess its impact on hospital CPB prevalence and the effectiveness of IPC measures. Patients were randomly selected for CPB rectal screening during Phases 1 and 3 and provided informed consent for inclusion.

**Results:**

The mBCT significantly shortened the mTAT and TPR compared to standard approaches (<24 h vs. 4d, p < 0.001), showing moderate sensitivity [54.6% (IC95% 45.2–63.7)] and high specificity [99.8% (IC 95% 99.3–100)]. The IPC intervention guided by the mBCT reduced CPB prevalence in general wards (8.1% vs. 13.8%, p = 0.006).

**Conclusion:**

The implementation of the NA reduced mTAT with high sensitivity, while the mBCT also contributed to reducing TPR with high specificity. Integrating the NA and mBCT into IPC protocols led to a decrease in CPB rectal carriage prevalence in general wards, underscoring their diagnostic, epidemiological and thus IPC benefits.

## 1 Introduction

Antimicrobial resistance represents an escalating global health crisis, with projections indicating that the previously estimated 10 million deaths per year by 2050 may now be outdated (O’Neill, 2016; Antimicrobial Resistance Collaborators, 2022). This phenomenon, driven largely by the misuse and overuse of antimicrobial agents, poses a significant threat not only to individual health but also to the efficacy of modern medicine as a whole. Particularly, carbapenemase-producing bacteria (CPB) are of grave concern due to their enzymatic ability to hydrolyze carbapenems, effectively removing broad-spectrum therapeutic options among beta-lactam antibiotics. These bacteria often carry additional resistance genes, further limiting treatment options. Their rapid spread, exacerbated during the COVID-19 pandemic, has become a global challenge, with significant increases noted in Latin America and the Caribbean, including our hospital (Thomas et al., 2022; Castro et al., 2022).

Contact precautions are an effective infection prevention and control (IPC) tool for controlling CPB dissemination. However, it carries drawbacks such as higher costs and reduced bed availability (Solter et al., 2018). Optimizing this process requires rapid identification of CPB carriers to reduce unnecessary isolation periods for non-carriers while ensuring prompt containment of actual carriers. Central to this task is microbiology stewardship, ensuring timely and accurate results to inform IPC strategies.

Several diagnostic approaches have been proposed to ensure the timely detection of rectal carriage, including commercially available real-time multiplex polymerase chain reaction (PCR) assays and in-house PCR protocols that can be performed directly on rectal swabs, as well as lateral flow immunoassays, which may be employed following a brief preincubation step. However, nowadays molecular-based or immunochromatography-based methods are out of reach for many centers, mainly due to unaffordable costs. In the absence of these rapid tests, we are left with long microbiological turnaround times (mTAT) which result in unnecessary hospital day-bed loss due to contact precautions or untimely initiation of contact precautions.

Blue-carba test (BCT) is a low-cost colorimetric assay extensively used in Argentina. It delivers results in under 2 h with a sensitivity greater than 90%, and a specificity close to 100%. However, these tests are optimized for use from bacterial colonies rather than directly from rectal swabs, requiring an incubation period of at least 24 h. Moreover, its main disadvantage is the difficulty in detecting Oxa-48-like variants (Pasteran et al., 2015; Maccari et al., 2024), which are not currently endemic in our hospital.

We conducted a prospective interrupted time series study from February 2022 to July 2023 in a 300-bed hospital from Argentina to evaluate the effectiveness of IPC measures guided by a novel algorithm (NA) for rectal swab processing, which incorporated a modified Blue-Carba test (mBCT), in reducing CPB rectal carriage prevalence in general wards. Additionally, we assessed the impact of this algorithm on microbiological turnaround time (mTAT) and time to positive results (TPR). Notably, at the start of the study, no CPB screening protocols were in place and CPB prevalence was unknown but was considered high due to local and regional trends in CPB infections.

## 2 Materials and methods

### 2.1 Study design

This study employed a multifaceted methodological approach, integrating experimental and quasi-experimental before-after designs into a prospective interrupted time series study structured in three phases. The methodological strategy described below was designed to validate the NA and the mBCT and to assess their effectiveness along with an IPC bundle in reducing CPB rectal carriage prevalence. The study adhered to the ORION statement’s guidelines for transparent reporting of outbreak reports and intervention studies of nosocomial infection.

The study was conducted in the general wards of a 300-bed tertiary-care hospital that serves as a referral center for the north-central region of the province of Santa Fe, Argentina. The hospital’s infrastructure is relatively new, as the institution relocated to a newly constructed facility in 2019. Patients in general wards are typically accommodated in two-bed rooms, unless they require contact precautions or other forms of isolation (e.g., respiratory or neutropenic precautions). The hospital has established policies for the management of patients colonized or infected with multidrug-resistant organisms; however, routine screening for rectal carriage of CPB was not implemented at the start of the study.

### 2.2 Experimental design: description and procedures of Phase 1

Phase 1 (P1, February 2022-July 2022) of the study included the set-up of the mBCT and the evaluation of the diagnostic performance following the design described below. In addition, during P1, the baseline prevalence of CBP rectal carriage was determined.

**FIGURE 1:**
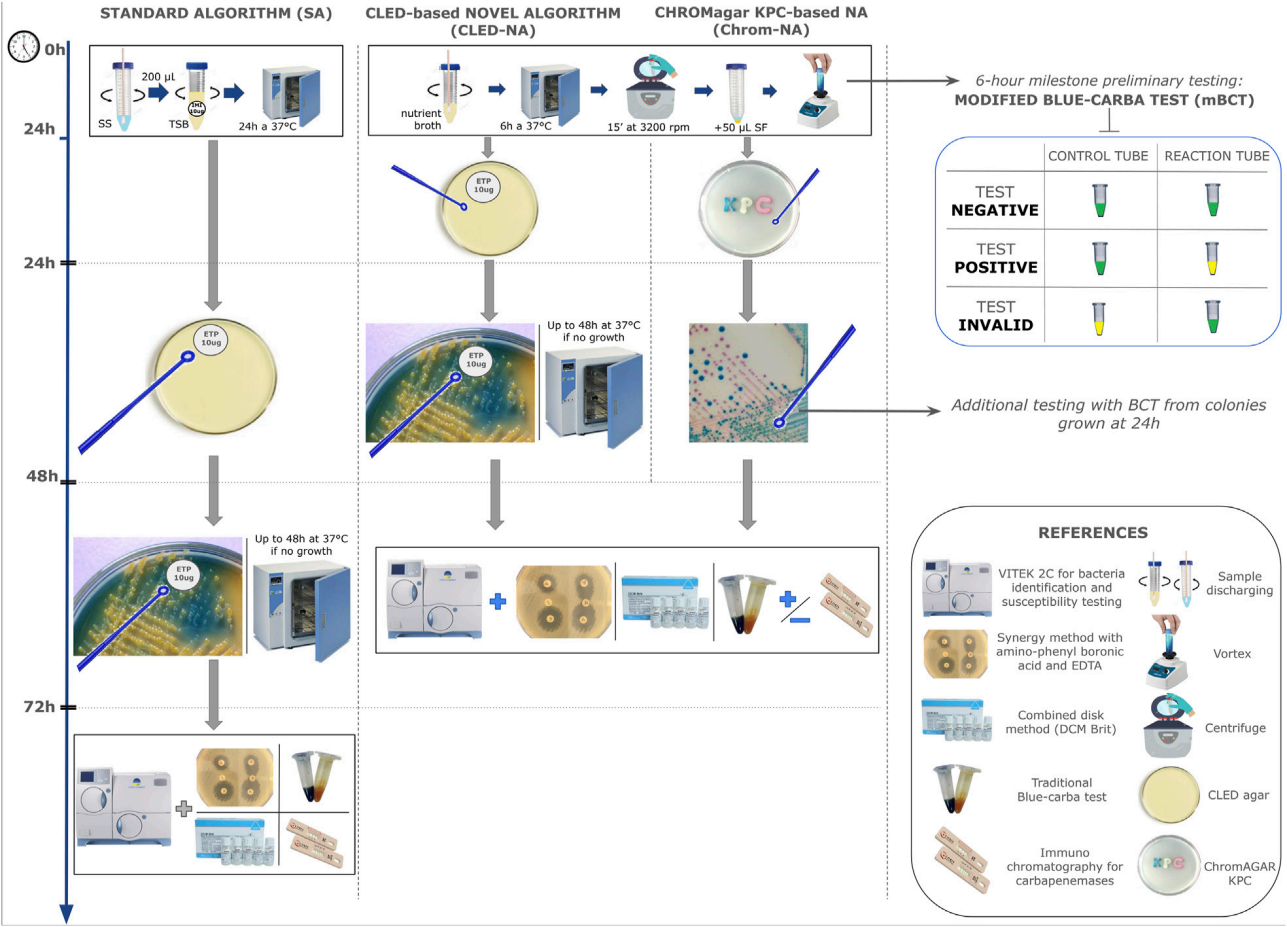
Diagnostic flowchart depicting the compared algorithms. Bench work time-line (in hours) on the left. Each block illustrates the step by step for each procedure (Standard Algorithm, CLED-NA and Chrom-NA). The panel on the left depicts additional tests performed to the samples as indicated. For more details, see descriptions in the text.

The microbiological techniques used in the experimental design during P1 were performed as follows. Two swabs were collected per patient. In all cases, patients were swabbed in a lateral decubitus position and a sterile cotton swab was inserted into the anal canal until it reached the recto-anal angle. The swab was then rotated 180° and removed. Afterwards, Swab 1 was processed following the standard algorithm (SA), while Swab 2 was processed following the NA, as shown in Figure 1 and briefly described below.

In the SA, Swab 1 was (i) discharged by rotation in a tube with 5 mL of tryptic soy broth with a 10 μg imipenem disc, which was then incubated for 18–24 h at 37 °C; and (ii) after incubation, 100 μL of this broth was subcultured onto a cysteine lactose electrolyte deficient (CLED) agar plate, with a 10 μg ertapenem disc (Figure 1).

Meanwhile for the NA, each swab was (i) rotated and rubbed against the base of a conic tube with 2 mL nutrient broth; (ii) incubated for 6 h at 37 °C and centrifuged for 15 min at 3,200 rpm; (iii) the supernatant was discarded and 50 μL of 0.9% NaCl was added, after which it was homogenized in a vortex; (iv) 50 μL was reserved for mBCT and the bottom of the conical tube was swabbed and discharged onto a CLED plate with a 10 μg ertapenem disc (Figure 1).

The mBCT was adapted from the original protocol as follows (Pasteran et al., 2015; Pires et al., 2013). Briefly, (i) three sterile Eppendorf tubes were used per reaction: reaction tube, control tube N° 1, and control tube N° 2, in addition to having positive and negative controls with known *Enterobacterales* strains; (ii) 50 μL of the sample was mixed in the reaction tube with 100 μL of 0.04% bromothymol blue aqueous solution and 0.1 mmol/L ZnSo_4_ supplemented with 9 mg/mL of commercial imipenem, adjusted to a final pH of 10.7; (iii) control tube N° 1 lacked imipenem and control tube N° 2 lacked the bacterial sample; (iv) the contents of the tubes were homogenized and incubated at 37 °C for a maximum of 2 h; (v) a positive result was indicated by a green-yellow color change in the reaction tube as shown in Figure 1, with no change in control tubes.

In both cases, the CLED plates, once inoculated, were incubated for up to 48 h at 37 °C, and then the colonies that developed around the ertapenem disk were studied. Identification and antimicrobial susceptibility testing were carried out using the Vitek 2C automated system (Biomérieux, Marcy-l'Etoile, France). Carbapenemase activity was confirmed using the double disk synergy test or by the combined disk method (DCM Brit) following protocols and interpretation criteria established by CLSI M100 (as updated in 2023, http://www.clsi.org) (Pasteran et al., 2011; Celenza et al., 2018; Arakawa et al., 2000; Pournaras et al., 2013).

Carbapenemase confirmation for selected samples was performed using a multiplex PCR to detect *bla*_KPC_, *bla*_NDM_, *bla*_IMP_, *bla*_VIM_, and *bla*_OXA-48-like_ genes (http://antimicrobianos.com.ar/wp-content/uploads/2024/03/Carbapenemases-Multiplex-qPCR-English.pdf). Also, a multiplex PCR to detect *bla*_CTX-M_, *bla*_PER_ ESBL and *bla*_CMY_ plasmid-borne AmpC was set up (http://antimicrobianos.com.ar/wp-content/uploads/2023/04/Deteccion-Mpx-CTX-M_PER_CMY.English.pdf). Amplification products were separated by electrophoresis in a 1% agarose gel and visualized with SYBR Safe (Thermo-Fisher Scientific, United States) in a molecular imager Gel Doc^TM^ XR+ using the Image Lab^TM^ Software (BioRad, United States). A 1 kb molecular marker (Invitrogen^TM^, Thermo Fisher Scientific, United States) and a positive control for each gene tested was run in the same gel. Only samples from the comparison between SA and NA were tested for carbapenemases. Immunochromatography was performed to confirm *bla*_OXA-48-like_ in cases where PCR was not done.

Later, the CLED-based NA (CLED-NA) was compared to a CHROMagar^TM^ KPC-based NA (Chrom-NA). As shown in Figure 1, procedures for the NA were similar to the ones already described. In addition to being paired with a mBCT (ChromNA-mBCT), in the Chrom-NA a second BCT was performed on isolates grown at 24 h.

### 2.3 Quasi-experimental before-after design: description of Phase 2 and 3

During P1 (February–July 2022) and P3 (February–July 2023), patients were prospectively selected using a computer-generated random number sequence to estimate the prevalence of CPB. Inclusion criteria for the prevalence assessment included age >14 years, admission to general medical or surgical wards, and provision of informed consent. Swabs were performed 2–3 times per week in a rotating fashion between general surgery and internal medicine wards. On each sampling day, 10 patients were randomly selected from the ward using simple randomization, and rectal swabbing was conducted after obtaining informed consent.

Before P2, no screening protocol por CPB rectal carriage was in place. In P2 (August 2022–January 2023), the intervention began with staff training and algorithm communication to all sectors involved. Subsequently, targeted screening of at-risk patients was implemented. High-risk patients for CPB colonization—with Intensive Care Units (ICU) stays in the past 6 months, hospitalizations over 7 days in the last month, or transfers from other hospitals—were swabbed and preventively isolated in single rooms with glove and gown precautions. Patients on broad-spectrum antibiotics were swabbed every 7 days, while the others were swabbed every 14 days. Additionally, those who shared a room for over 24 h with a CPB carrier were also swabbed and isolated. Identified carriers were promptly isolated, all involved personnel were informed, and contact tracing was conducted. Hand hygiene adherence was monitored throughout P2.

During P2, the research team conducted systematic observations to assess hand hygiene adherence. These observations were performed randomly across various time frames, utilizing standardized questionnaires provided by the National Program for Epidemiology and Control of Healthcare-Associated Infections (VIHDA, by its Spanish acronym). Prior to data collection, observers received targeted training on the methodology to ensure consistency and reliability in data recording. Adherence to hand hygiene was evaluated based on the opportunities defined by the World Health Organization’s (WHO) “Five Moments for Hand Hygiene” framework, with each moment representing a distinct opportunity for compliance by the observed healthcare professionals.

During P3, staff continued intervention measures, and post-intervention CPB prevalence was assessed.

### 2.4 Comparisons of the experimental phase

After the experimental phase, the comparisons performed were: (i) the sensitivity, negative predictive value and mTAT - defined as the time from the reception of the sample at the clinical Microbiology Laboratory to the issuance of the final microbiological report - between the SA and the NA, (ii) the sensitivity, specificity, negative predictive value, positive predictive value, positive likelihood ratio and negative likelihood ratio of the mBCT compared to the NA, and the change in TPR - defined as the time from receipt at the Microbiology Laboratory to the alert of positivity issued to the treating physician, (iii) *a posteriori*, we decided to assess the difference in sensitivity when incubating samples for 24 h compared to an extended period of up to 48 h, (iv) the sensitivity, mTAT and TPR between the Chrom-NA and the CLED-NA.

### 2.5 Analysis of the intervention effectiveness

For the analysis of the effectiveness of the intervention, the primary analysis of interest was the comparison of CPB prevalence between the P3 (post-intervention) and the P1 (pre-intervention). Secondarily, we aimed to identify whether there was a significant change in the prevalence trend after the intervention in a time-series analysis.

We conducted a sensitivity analysis to verify if the difference in the primary outcome persisted when considering only the first sample collected for each patient. Given that the ICU typically has the highest rate of CPB infections, indicating a probable higher rate of rectal carriage, we intended to adjust the time-series analysis by using ICU density as a covariate. ICU density was defined as the number of ICU patient-days during a period divided by the total count of patient-days in adult wards for the same period.

### 2.6 Statistical analysis

A sample size of 435 swabs per group (870 total) was calculated using OpenEpi, based on a two-sample comparison of proportions. The calculation assumed a baseline CPB prevalence of 10% and aimed to detect a 50% relative reduction (to 5% post-intervention), with 80% statistical power and a two-sided alpha of 0.05.

CPB rectal carriage prevalence was calculated using the number of rectal swabs with CPB isolation as the numerator, and the total number of rectal samples collected during the study period as the denominator. Prevalence was expressed as the percentage of CPB-positive individuals among those swabbed.

The distribution of numerical results was studied using the Kolmogorov-Smirnov test. Normally distributed results were presented as mean ± SD, and were statistically analyzed using two-tailed t-tests. Results not meeting the normality condition were presented as median ± interquartile range and analyzed with Mann-Whitney’s U. Qualitative variables were expressed as percentages (%) and differences between proportions were evaluated using χ2 tests. Concordance was assessed using Cohen’s kappa. A p-value <0.05 was considered significant (using two-tailed tests). For multivariate analysis, a binary logistic regression was conducted to predict CPB rectal carriage with variables that showed association in the univariate analysis at a p < 0.1 significance level. The primary analysis, aimed at assessing the intervention’s effectiveness, was conducted through a comparison of proportions.

Regarding hand hygiene compliance, it was calculated as the proportion of performed hand hygiene actions over the total number of observed opportunities, in accordance with the WHO “Five Moments for Hand Hygiene” framework. Compliance rates were expressed as percentages. No statistical comparisons were performed, as these data were collected only during the intervention period and not in the pre- or post-intervention phases.

General statistical analyses were conducted using SPSS Statistics version 27 (IBM). The interrupted time-series analysis was carried out on Google Colaboratory using Python and the statsmodels library to implement an autoregressive integrated moving average (ARIMA) model.

The ARIMA(1,0,0) model was selected to account for serial correlation in the outcome since a preliminary segmented linear regression model showed residual autocorrelation (as indicated by the Durbin-Watson statistic), precluding the use of ordinary least squares. The dependent variable was the prevalence of CPB rectal carriage, calculated as the proportion of positive rectal swabs among all patients screened during each interval.

Although both the pre-intervention and post-intervention phases spanned six calendar months, each was divided into eight equal intervals to generate a sufficient number of time points for time-series modeling. A binary variable (INTERVENTION) was created to represent the implementation of the intervention and was included as an exogenous regressor. This approach allowed for the estimation of an immediate level change in CPB prevalence following the intervention, while accounting for potential autocorrelation in the data.

To estimate the counterfactual scenario—that is, the expected trend in the absence of intervention—a second ARIMA(1,0,0) model was fitted using only the pre-intervention data. Forecasts from this model were projected across the post-intervention period and compared with the observed values to assess the direction and magnitude of the intervention’s impact.

Seasonal modeling was explored, but the limited number of time points in each phase precluded the use of seasonal ARIMA models. ICU patient density was initially included as a covariate but was excluded from the ARIMA model due to lack of statistical significance in the univariate analysis.

### 2.7 Ethical considerations

This trial received approval from the Scientific and Teaching Committee of JB Iturraspe Hospital and obtained ethics approval from the Provincial Ethics Committee of Santa Fe. The study was conducted in accordance with the Council for International Organizations of Medical Sciences and the International Council of Harmonization Good Clinical Practices guidelines and adhered to the Declaration of Helsinki, the Belmont Report and the Nuremberg Code. Patient information was kept confidential, and patients were included in the study only after providing informed consent. In accordance with Argentine legislation (Civil and Commercial Code, Article 26), adolescents aged 14 and older may provide informed consent for low-risk research. As the study posed minimal risk, the ethics committee approved enrollment of participants over 14 years without requiring parental consent.

The quasi-experimental approach, chosen based on considerations from previous publications (Harris et al., 2004; Harris et al., 2005; Alsaggaf et al., 2018), was selected due to ethical concerns about using a control group.

## 3 Results

### 3.1 Validation of the novel algorithm

We tested 228 duplicate swabs (Table 1). Among the studied population, 18.9% (n = 43) rendered 68 isolates positive for CPB by either method (SA and/or NA). These included 58 *Enterobacterales* isolates and 10 non-fermentative bacilli. In two patients (M302 and M404, view Table 1), two distinctive carbapenemase-producing *Enterobacterales* were isolated from the same sample. *Klebsiella pneumoniae* was the most frequently isolated species (66.2%, n = 45) (Table 1; Supplementary Table S1).

**TABLE 1:** Comparison of isolates between the standard algorithm and the novel algorithm obtained during P1.

Isolate ID	Standard algorithm (SA)			Novel algorithm (NA)			Isolate concordance	Carriage concordance
	Microorganism	Phenotype	CBP gene	Microorganism	Phenotype	CBP gene		
M200	-	-	-	*K. pneumoniae*	KPC	KPC	No	No
M203	-	-	-	*K. pneumoniae*	MBL	NDM	No	No
M206	-	-	-	*K. pneumoniae*	MBL	NDM+ CTX-M	No	No
M208	*K. pneumoniae*	MBL	NDM+ CTX-M	*K. pneumoniae*	MBL	NDM+ CTX-M	Yes	Yes
M301	*P. putida*	MBL	VIM	*A. lwoffii*	MBL	NDM	No	Yes
M302	*E. cloacae*	MBL	NDM+ CTX-M	*E. cloacae + E. coli*	MBL/MBL	NDM+ CTX-M/NDM	No	Yes
M304	*P. putida*	MBL	VIM	-	-		No	No
M307	*K. pneumoniae*	KPC	KPC	*K. pneumoniae*	KPC	KPC	Yes	Yes
M401	*K. pneumoniae*	KPC	KPC	*K. pneumoniae*	KPC	KPC	Yes	Yes
M404	*E. coli + K. pneumoniae*	MBL (both)	NDM/NDM + CTX-M	*K. pneumoniae*	MBL	NDM+CTX-M	No	Yes
M502	*E. coli*	MBL	NDM + PER	*E. coli*	MBL	NDM + PER	Yes	Yes
M503	*C. freundii*	MBL	NDM + PER	-	-	-	No	No
M600	*K. pneumoniae*	MBL	NDM+ CTX-M	*K. pneumoniae*	KPC	KPC	No	Yes
M604	*K. aerogenes*	KPC	KPC	-	-	-	No	No
M607	*S. maltophilia*	MBL	ND	*E. coli*	MBL	NDM+ CTX-M	No	Yes
M609	*E. cloacae*	KPC	KPC	*K. pneumoniae*	KPC	KPC	No	Yes
M814	*K. pneumoniae*	KPC	KPC	*K. pneumoniae*	KPC	KPC	Yes	Yes
M826	*C. indologenes*	MBL	ND	-	-	-	No	No
M832	*P. alcaligenes*	MBL	VIM	-	-	-	No	No
M836	-	-		*K. pneumoniae*	MBL	NDM	No	No
M837	-	-		*S. marcescens*	KPC	KPC	No	No
M838	*E. coli*	MBL	NDM	-	-		No	No
M846	*P. alcaligenes*	MBL	VIM	-	-		No	No
M854	*K. pneumoniae*	MBL	NDM	*K. oxytoca*	MBL	NDM	No	Yes
M858	*K. pneumoniae*	KPC	KPC + CTX-M	*K. pneumoniae*	KPC	KPC + CTX-M	Yes	Yes
M875	*K. pneumoniae*	KPC	KPC	*K. pneumoniae*	KPC	KPC	Yes	Yes
M884	*S. paucimobilis*	MBL	ND	-	-	-	No	No
M885	-	-	-	*K. pneumoniae*	MBL	NDM + CTX-M	No	No
M888	*K. pneumoniae*	KPC	KPC + NDM	-	-	-	No	No
M892	*A. lwoffii*	MBL	NDM	-	-		No	No
M906	-	-	-	*K. pneumoniae*	KPC	KPC	No	No
M910	*P. putida*	MBL	VIM	-	-		No	No
M913	-	-	-	*K. pneumoniae*	KPC	KPC	No	No
M928	*K. pneumoniae*	MBL	NDM + CTX-M	*K. pneumoniae*	MBL	NDM + CTX-M	Yes	Yes
M932	*K. pneumoniae*	MBL	NDM	*K. pneumoniae*	MBL	NDM	Yes	Yes
M933	-	-	-	*K. pneumoniae*	MBL	NDM + CTX-M	No	No
M938	*K. pneumoniae*	MBL	NDM + CTX-M	*K. pneumoniae*	MBL	NDM + CTX-M	Yes	Yes
M946	*K. pneumoniae*	KPC	KPC	*K. pneumoniae*	KPC	KPC	Yes	Yes
M947	*K. pneumoniae*	MBL	NDM + CTX-M	*K. pneumoniae*	MBL	NDM + CTX-M	Yes	Yes
M951	*K. pneumoniae*	MBL	NDM + CTX-M	*K. pneumoniae*	MBL	NDM + CTX-M	Yes	Yes
M953	*K. pneumoniae*	KPC	KPC	*K. pneumoniae*	KPC	KPC	Yes	Yes
M954	*K. pneumoniae*	KPC	KPC	*K. pneumoniae*	KPC	KPC	Yes	Yes
M955	*K. pneumoniae*	MBL	NDM + CTX-M	*K. pneumoniae*	MBL	NDM + CTX-M	Yes	Yes

CBP, carbapenemase; KPC, *Klebsiella pneumoniae* carbapenamase; MBL, metallo-beta-lactamase; NDM, New-Delhi metallo-beta-lactamase; ND, Not determined; “-”, negative. Isolate concordance, isolation of the same species and carbapenemase phenotype by SA and NA. Carriage concordance: concordance of the results by the SA and NA dichotomized in positive or negative for carriage.

Among the *Enterobacterales* isolates, 58.6% (n = 34) were confirmed as *bla*_NDM_ and 43.1% (n = 25) as *bla*_KPC_. In one patient (M888), one multiple carbapenemase-producing (KPC and NDM) *K. pneumoniae* was isolated (Table 1). Of these, 44.8% (n = 26) also carried ESBL genes [88.5% (n = 23) *bla*_CTX-M_, and the rest *bla*_PER_]. Of note, no *bla*_VIM_ or *bla*_IMP_ carbapenemases were detected among these samples (Table 1).

Among the 10 non-fermentative bacilli, *P. putida* and *P. alcaligenes* expressed *bla*_VIM_ and *A. lwoffii bla*_NDM_, without ESBL expression (Table 1; Supplementary Table S1).

In terms of CPB carriage status, there was moderate concordance between the two algorithms (*κ* 0.65; p < 0.001; 91.2%, 185 negative and 23 positive cases). However, the SA identified 11 positive cases that the NA missed, and in turn the NA identified 9 additional positive cases not detected by the SA. The sensitivity and negative predictive value of both algorithms were not significantly different (sensitivity 79.1% vs. 77.4%; negative predictive value 95.4% vs. 94.4%).

The SA had a longer mTAT by 1 day (4 vs. 3, p < 0.001), and required an additional 5 days of laboratory work for each positive result (29 vs. 24). The subgroup of positive samples also experienced a longer mTAT of one additional day (5 vs. 4, p < 0.001).

To compare 24-hour vs. 48-hour incubation times, 565 samples were processed with the NA. Of 70 CPB-containing samples, 67 (95.7%) showed growth at 24 h. After 48 h, 12 additional samples exhibited growth, but 9 (75%) were non-CPB.

### 3.2 mBCT validation against culture (by NA)

The mBCT, as a preliminary testing method for CPB, was validated against a culture method processed by the NA. A total of 1120 samples were processed, revealing an overall CPB prevalence of 9.6% (n = 108) in general wards (Supplementary Table S2). KPC phenotype was exhibited by 63.9% (n = 69), whereas only two isolates expressed multiple carbapenemases (KPC+MBL), and another two were identified as OXA-48 producers. The mBCT showed moderate sensitivity and high specificity (Table 2). No significant differences were observed when the analysis was conducted separately for KPC and MBL phenotypes. The implementation of mBCT significantly reduced median TPR (<24 h vs. 4 days, p < 0.001).

**TABLE 2:** Diagnostic performance of the mBCT with CLED-NA as the reference.

Performance metrics	Point estimate	95% confidence interval
Sensitivity	54.60%	45.2–63.7
Specificity	99.80%	99.3–100
Positive predictive value	96.70%	88.8–99.1
Negative predictive value	95.80%	94.5–96.8
Positive likelihood ratio	276	-
Negative likelihood ratio	0.45	-

CLED, Cystine lactose electrolyte deficient agar; NA, Novel algorithm

### 3.3 mBCT using Chromagar KPC plates and complementary direct BCT

The performance of Chrom-NA vs. CLED-NA was compared in samples from 523 patients by streaking on both plates. As a result, 55 patients (10.5%) tested positive by either method, yielding 97 CPB isolates (Supplementary Table S3). The concordance between the two methods to detect carbapenemase production was moderate to high (k 0.85, p < 0.001). The Chrom-NA demonstrated superior sensitivity (98.2% vs. 78.2%, p = 0.001) and higher NPV (99.8% vs. 97.5%, p = 0.002). Moreover, incorporating a direct BCT, where feasible, from colonies grown at 24 h on Chromagar KPC plates also allowed the Chrom-NA to reduce TPR for positive results compared to the NA (2 days vs. 4 days, p < 0.001).

The Chrom-NA yielded 91.2% (n = 52) of results at 24–48 h, and the rest took longer. The Chrom-NA-mBCT, which also included a mBCT directly from rectal swabs (Figure 1), detected 42.1% (n = 24) of CPB at 6 h, 49.1% (n = 28) within 24–48 h and the rest took longer.

### 3.4 Effectiveness of a mBCT-guided algorithm for IPC

Among the 735 participants, median age was 48 years (IQR 33–58) and male sex predominated (56.3%, n = 414), both categories without difference between periods. During P3 there was a higher rate of patients with a previous ICU stay [22.7% (n = 80) vs. 15.4% (n = 58), p = 0.012] and a longer previous in-hospital stay [7 days (IQR 4–13) vs. 6 days (IQR 6–12), p < 0.001]. Patients with CPE carriage showed no difference regarding age or sex, but had more frequently a previous ICU stay [60.3% (n = 47) vs. 14.0% (n = 91), p < 0.001] and a longer previous in-hospital stay [12.5 days (IQR 7–37) vs. 6 (IQR 3–11), p < 0.001]. No significant correlation was found in the univariate analysis between monthly CPB prevalence and ICU patient density.

The intervention significantly reduced CPB prevalence [P3 8.1% (n = 29) vs. P1 13.8% (n = 52), p = 0.006]. In the planned sensitivity analysis, the observed reduction in CPB prevalence was maintained after excluding samples that were collected from the same patients on different occasions (8.1% vs. 12.9%, p = 0.02).

In the multivariate analysis, a previous ICU stay (OR 9.96 95% CI 5.74–17.3, p < 0.001) and previous in-hospital length of stay (1.01 95% CI 1.01–1.02, p = 0.023) increased chances of CPE carriage and hospitalization during P3 decreased them (OR 0.334 95% CI 0.191–0.584, p < 0.001) (735 patients included, model’s p < 0.001, Nagelkerke’s R^2^ = 0.253).

The ARIMA(1,0,0) model, which included the intervention phase as an exogenous regressor, estimated an immediate level change in CPB prevalence of −0.059 (95% CI: −0.118 to 0.000; p = 0.051) following the intervention. The model included a constant baseline prevalence level (intercept = 0.1395) and an autoregressive term (AR(1) coefficient = −0.2832; p = 0.661), indicating no significant autocorrelation in the outcome series. Model diagnostics showed good overall fit and no evidence of autocorrelation, heteroscedasticity, or non-normal residual distribution (Supplementary Table S4).

To assess the potential impact of the intervention, a counterfactual forecast was generated by fitting the same ARIMA(1,0,0) model to the pre-intervention data only. Predicted values for the post-intervention period were consistently higher than the observed prevalence, suggesting a downward shift potentially attributable to the intervention (Supplementary Figure S1).

**FIGURE 2:**
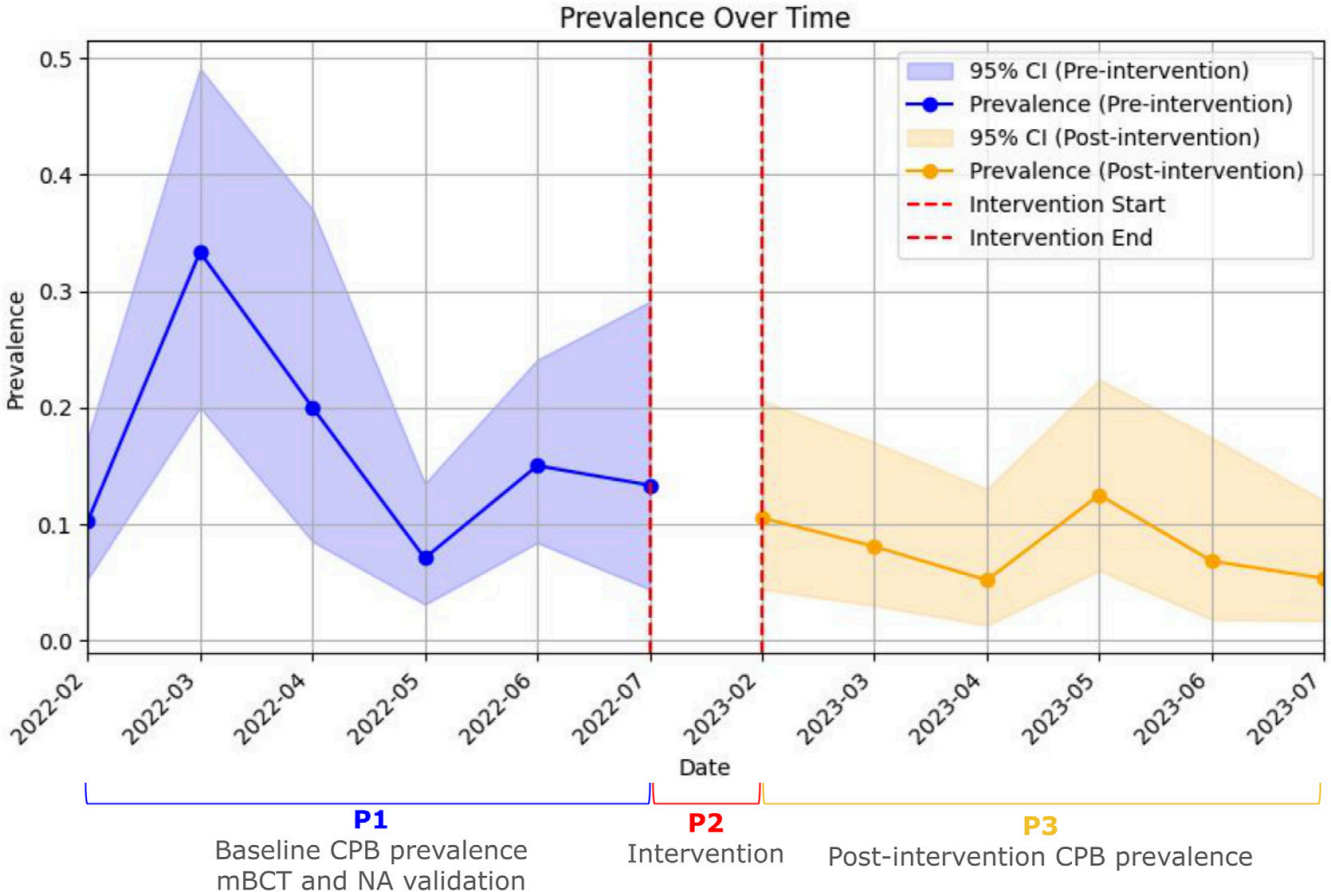
Interrupted time-series analysis of carbapenem-producing bacteria rectal carriage prevalence before and after the Intervention. Monthly prevalence of rectal colonization with carbapenemase-producing bacteria over the study period. The solid lines represent observed prevalence during the pre-intervention and post-intervention phases. Shaded areas indicate 95% confidence intervals. Vertical dashed red lines mark the beginning and end of the intervention phase. Each phase (pre- and post-intervention) was divided into eight equal-length intervals to generate sufficient data points for time-series modeling. CI: Confidence interval; P1: Phase 1; P2: Phase 2; P3: Phase 3; CPB: carbapenemase-producing bacteria.

A descriptive time-series plot displaying monthly CPB prevalence with 95% confidence intervals and the timing of the intervention is presented in Figure 2. Visually, a baseline downward trend was observed, as well as a seasonal pattern (Supplementary Figure S2). However, seasonality could not be studied due to the limited number of data points which prevented formal modeling with seasonal ARIMA.

### 3.5 Assessment of hand hygiene compliance during the intervention

A total of 161 healthcare professionals were observed during 405 hand hygiene opportunities. The overall compliance rate was 25.4% (n = 103), with 46.6% (n = 42) of instances demonstrating the correct hand hygiene technique. Detailed hand hygiene compliance rates and the proportion of correct technique across subgroups are presented in Table 3.

**TABLE 3:** Hand hygiene compliance and technique adequacy.

Category		Total observations (n = 405) [%(n)]	Compliance rate (n = 405) [%(n)]	Correct technique (n = 90) [%(n)]
Profession	Physicians	32.8% (133)	33.8% (45)	79.1% (34)
	Nurses	39.3% (159)	28.3% (45)	8.33% (3)
	External Personnel	26.2% (106)	10.4% (11)	45.5% (5)
Moments of hand hygiene	Before patient contact	35.5% (144)	15.3% (22)	63.2% (12)
	Before aseptic tasks	3.70% (15)	20.0% (3)	50.0% (1)
	After risk of exposure to biological fluids	5.19% (21)	57.1% (12)	20.0% (2)
	After patient contact	26.4% (107)	30.8% (33)	53.3% (16)
	After contact with patient’s surroundings	29.1% (118)	28.0% (33)	37.9% (11)
Overall adherence		100% (405)	25.4% (103)	46.6% (42)

## 4 Discussion

Our findings support BCT as an optimal candidate for modification due to simplicity, minimal cost, and widespread use in Argentine laboratories. The proposed algorithm enabled reporting of 50% of positive results within 6 h and all results within 24 h, a significant improvement over the SA, which required at least 72 h. Furthermore, IPC measures guided by rectal swabs, processed with the proposed algorithm, effectively reduced CPB rectal carriage prevalence in general wards.

At the beginning of the study, the CPB rectal carriage prevalence in general wards was unknown, with very little data available from regional studies. However, it was suspected to be high, given the reported incidence of CPB infections. This baseline information was not only crucial for initiating this study, but also held significant epidemiological relevance, as rectal carriage is a recognized factor contributing to CPB dissemination. We found a high baseline of CPB rectal carriage prevalence. We found *bla*_NDM_ predominance, which was to be expected based on reports from the National and Regional Reference Laboratory in Antimicrobial Resistance (NRRLAR) - ANLIS “Dr Carlos G. Malbrán”, which highlight its predominance among carbapenemases nationwide. However, *bla*_KPC_ had previously been identified as the most common carbapenemase in infections caused by CPB (Castro et al., 2022).

The NA outperformed CDC-recommended SA by reducing mTAT. This reduction in mTAT by 1 day was due to the removal of the 24-hour incubation in tryptic soy broth with carbapenem needed in the SA. The NA also has the advantage of being suitable for settings without access to chromogenic media, as was initially the case for our hospital. The 24-hour incubation proved as effective as 48 h, further reducing overall mTAT.

The NA also allowed the coupling of a mBCT at 6-hours of sample receipt at the microbiology laboratory. The integration of the mBCT reduced TPR by providing rapid and highly specific results, with moderate sensitivity, ensuring no cases were missed due to the concurrent use of solid medium cultures. The shorter TPR facilitated quicker isolation and contact tracing. Finally, the Chrom-NA-mBCT further reduced TPR.

Later, with the introduction of CHROMagar^TM^ KPC at our center, a comparative analysis with the CLED-NA revealed the Chrom-NA’s higher sensitivity and its capability for direct BCT on colony isolates, significantly shortening the TPR once again. Regarding the higher sensitivity of the Chrom-NA compared to the CLED-NA, it must be pointed out that results regarding the sensitivity of chromogenic media in the literature are diverse. While specificity is usually higher than regular agar plates with carbapenem disks, sensitivity has been reported to be equal (Vrioni et al., 2012), lower (poster sessions, not published) or higher (Errecalde et al., 2012; Samra et al., 2008).

The final algorithm performance must be compared with other platforms for rapid diagnosis of CPB rectal carriage, both molecular or immunochromatography-based. For instance, multiple platforms based on DNA detection have been tested to be used directly from rectal swabs, such as Xpert Carba-R (Cepheid, Sunnyvale, United States), BD MAX Check-Points CPO Assay (BD Biosciences, Franklin Lakes, United States) and Amplidiag^®^ CarbaR+VRE (Mobidiag, Espoo, Finland). These platforms demonstrated high sensitivity and specificity. However, their high sensitivity also makes them prone to detecting very low DNA loads from non-viable cells or due to cross-contamination, which can complicate the interpretation of results. (Cury et al., 2020; García-Fernández et al., 2022; Girlich et al., 2020; Nadji et al., 2019; Moore et al., 2017; Nijhuis et al., 2013). Moreover, their limited availability in certain jurisdictions and the high costs per reaction further hinder their implementation, particularly in high-capacity hospitals with substantial CPB screening demands. Even when these platforms are accessible, maintaining a consistent supply of reagents over time remains challenging due to cost-related constraints.

Among lateral-flow immunochromatographic methods, OKN K-SeT and Resist-4 O.K.N.V. K-SeT kits (Coris BioConcept, Gembloux, Belgium) have shown high sensitivity and specificity to detect CPB directly from rectal swabs after a short incubation period, using selected samples and spiked mock rectal swabs. NG-Test CARBA 5 (NG Biotech, Guipry-Messac, France) has also been evaluated after a short incubation period, with the same results. This has been further integrated in a SPID platform (BL-DetecTool, NG Biotech, Guipry-Messac, France) showing similar results. However, this last tool showed variable sensitivities (66.6%–100%) in real world settings (Fauconnier et al., 2019; Gallah et al., 2021; Wang et al., 2023; Vasilakopoulou et al., 2021; Volland et al., 2022; Fernande et al., 2024). Despite their promising performance, these methods face significant limitations in terms of affordability and availability, particularly in regions like South America, where access to such tools can be restricted by high costs and logistical challenges.

To support the implementation of Chrom-NA-mBCT in our hospital, we calculated the costs of the mBCT compared to other available methodologies. These initial calculations, pending a comprehensive cost-effectiveness study, showed that the mBCT may have reduced expenses compared to other testing strategies, particularly when considering the cost of rectal swabs and culture media. For instance, immunochromatography costs up to USD 18 per sample, and multiplex PCR costs USD 54 per sample, whereas mBCT costs only USD 0.02 per sample. Based on the 642 protocol-mandated rectal swabs during the intervention period, the use of mBCT would yield cost savings of USD 11,543.16 and USD 34,655.16, respectively, when compared to the adoption of immunochromatography or multiplex PCR methodologies.

The mBCT-guided IPC algorithm halved CPB rectal carriage prevalence in general wards. Multivariate analysis showed that patients hospitalized after its implementation had a two-thirds lower likelihood of CPB rectal carriage. Our study illustrates the impact of a surveillance- and contact precautions-centered intervention in a Latin American tertiary-care center, whereas previous studies showed variable results, with a high heterogeneity that precluded meta-analysis (Hernández-García et al., 2021; Hagiya and Otsuka, 2023; Gagliotti et al., 2014; Schwaber et al., 2011; Lane et al., 2021; Viale et al., 2015; Spyridopoulou et al., 2020; Ben Natan et al., 2023; Otter et al., 2020).

Regarding compliance with IPC measures during the intervention, we observed very low rates of hand hygiene, significantly below the already low national averages reported in the National Study on Hand Hygiene Prevalence in Argentina 2023. While the national average for hand hygiene adherence in non-critical care units is 56.2%, our observations showed a rate of 25.4% when combining hand hygiene both before and after patient contact. In contrast, the percentage of correct hand hygiene technique was only slightly below the national average (46.6% vs. 62.4%). This limited adherence may have compromised the effectiveness of the intervention, particularly in preventing cross-transmission and reducing CPB rectal carriage. It is also possible that hand hygiene adherence varied between healthcare personnel managing patients under contact precautions versus those under standard precautions, which may have influenced outcomes. Moreover, although hand hygiene constitutes the cornerstone of IPC, and gloving is intended to serve as a complementary measure, it is possible that in this context, gloving played a more substantial role in limiting the dissemination of CPB from identified carriers—especially in the setting of shared two-bed rooms. Although the number of observations was limited due to personnel constraints, the strength of the data lies in the randomized selection of opportunities and observer training. These findings suggest that the observed reduction in CPB prevalence might underestimate the full potential of the intervention, given the suboptimal adherence to key IPC measures such as hand hygiene.

With regard to contact precautions, appropriate precautions were consistently implemented for all cases in accordance with the study protocol, as this process was supervised by the study group. However, individual adherence to the use of gloves and gowns could not be thoroughly assessed due to personnel constraints, resulting in an insufficient number of observations to allow for statistical analysis.

During the study period, no significant changes were made to hospital policies regarding IPC or antibiotic stewardship. Moreover, no additional interventions targeting the prevention of CPB in general wards were implemented beyond those introduced as part of this study and the routine education of cleaning staff, as outlined in existing hospital policies.

A strength of this study was the use of random sampling to compare prevalence across two periods, preventing the artificial increase in post-intervention CPB prevalence due to higher sampling rates (Otter et al., 2020). However, the lack of a control group to assess CPB rectal carriage prevalence without the intervention limits the ability to rule out regression to the mean or maturation effects (Harris et al., 2004; Harris et al., 2005). However, this comparison was not made due to ethical concerns, given that international guidelines already recommend rectal screening for CPB and isolation of carriers (Magiorakos et al., 2017). Furthermore, the complexities involved in managing samples and notifications for two randomized sample groups in everyday practice also precluded the processing of one intervention group’s samples with SA and another’s with NA and mBCT in parallel.

Additionally, when an interrupted time series analysis was performed, it indicated a trend towards a decrease in prevalence that approached but did not reach statistical significance. Upon analyzing the model, it demonstrated an overall good statistical fit. However, there were specific limitations that could explain the failure to achieve statistical significance. First, the observation time points were the minimum required for the model to perform adequately. Second, an unexpected baseline downward trend in P1, possibly influenced by rectal screening, may have affected patient handling even without an intervention. Third, a certain degree of seasonality was observed. Although it did not align with specific months, this seasonality could suggest the presence of underlying cyclical processes. Unfortunately, these processes could not be adequately analyzed through seasonal ARIMA modeling. This inadequacy was due to the fact that, since this seasonality was unexpected, the number of time periods included was insufficient for comprehensive modeling.

Although external factors and a pre-existing downward trend cannot be excluded as plausible contributors to the observed reduction in CPB rectal carriage following the intervention, the study had two notable strengths that helped mitigate this risk. First, the intervention was implemented approximately 6 months after the last wave of the COVID-19 pandemic—a period characterized by increased ICU occupancy and other pressures that contributed to a rise in CPB infections. While some natural regression may have occurred post-pandemic, this must be interpreted in the broader epidemiological context of Argentina, and the province of Santa Fe in particular, where a sustained increase in CPB incidence has been documented. Second, although the results of the ARIMA analysis approached but did not reach statistical significance—possibly due to the aforementioned external influences—they nonetheless provide partial support for the primary analysis, which showed a reduction in the prevalence of CPB rectal carriage.

One potential limitation was the combination of a higher-than-expected baseline prevalence of CPB and lower-than-anticipated patient enrollment, which reduced the study’s statistical power to 70.4%. This reduction increased the risk of a Type II error—that is, failing to detect a true effect. However, this concern is mitigated by the fact that significant differences were observed following the intervention.

The performance of the Chrom-NA-mBCT must be interpreted within our hospital epidemiology, with KPC and NDM predominance, with fewer cases of Oxa-48-like and multiple carbapenemase producers. Moreover, most carbapenemases are expressed in *Enterobacterales*, with only a few cases of carbapenemase-producing non-fermenting bacilli. The impact of the intervention should also be considered within the context of several key factors: the absence of baseline screening for CPB rectal carriage, a high baseline prevalence of CPB, and low adherence to hand hygiene practices. Finally, given the bundled nature of the intervention, it is not possible to determine the extent to which the observed outcomes can be attributed to routine screening and isolation measures versus the microbiological enhancements that enabled more rapid identification of CPB rectal carriage. However, the bundled approach also represents a strength of the study, as the microbiological modifications enhanced the implementation of the intervention by facilitating two critical processes: the early de-isolation of non-carriers, thereby minimizing unnecessary bed occupancy, and the rapid isolation of carriers, thereby reducing the risk of further dissemination.

In conclusion, this study developed and validated an economical and efficient algorithm integrating rectal swab processing modifications with the BCT. The final algorithm, ChromNA-mBCT, significantly reduced both the mTAT and TPR, allowing for the identification of half of the carriers within 6 h of sample collection and the remainder within 24 h, with very high specificity. The intervention, guided by rectal swabs processed by this new algorithm, significantly reduced CPB rectal carriage prevalence in general wards by facilitating rapid identification, prompt isolation of CPB carriers and contact tracing. This is one of the few studies to systematically demonstrate effective strategies for reducing CPB prevalence outside outbreak settings. Additionally, it highlights the feasibility of implementing low-cost methods in both high- and low-resource healthcare settings, offering a model for broader application in endemic regions.

## Data Availability

The raw data supporting the conclusions of this article will be made available by the authors, without undue reservation.
