# Supplementary material for: Effectiveness of infection control measures informed by a modified Blue-Carba test in reducing rectal carriage of carbapenemase-producing bacteria in general wards: a prospective interrupted time series study
Source: Front Pharmacol. 2025 Sep 4;16:1584646. doi: 10.3389/fphar.2025.1584646 (PMC12443808; doi:10.3389/fphar.2025.1584646)
Supplement: Supplementary file 1 [file DataSheet1.docx]

**Online Supplement to:** Effectiveness of infection control measures informed by a modified Blue-Carba test in reducing rectal carriage of carbapenemase-producing bacteria in general wards: a prospective interrupted time series study

Maximiliano Gabriel Castro; Argarañá, Fernanda; Bernasconi, Carla; Margenet, Leticia; Amato, Ana Paula; Coduri Anthonioz Blanc, Joaquín Ignacio; Rottoli, Erwin Alexander; Protto, Manuel; Vicino, Macarena; Sadonio, María José; Galluccio, Federico Rafael; Musacchio, Héctor Mario; Pasterán, Fernando; Gómez, Sonia Alejandra.

**Table S1.** Distribution of CPB isolates, phenotypic and genotypic carbapenemases in 228 duplicate swabs in the comparison of NA vs SA.

| Species | % of isolates (n) | Phenotypic CBP | | Genotypic CBP | | | Genotypic ESBL | |
| --- | --- | --- | --- | --- | --- | --- | --- | --- |
|  |  | KPC [% (n)] | MBL [% (n)] | *blaKPC* | *blaNDM* | *blaVIM* | *bla*CTX-M | *bla*PER |
| *K. pneumoniae^#^* | 66.2% (45) | 48.9% (22) | 51.1% (23) | 48.9% (22) | 53.3% (24) | 0.00% (0) | 44.4% (20) | 0.00% (0) |
| *E. coli* | 8.82% (6) | 0.00% (0) | 100% (6) | 0.00% (0) | 100% (6) | 0.00% (0) | 33.3% (1) | 33.3% (2) |
| *E. cloacae* | 4.41% (3) | 33.3% (1) | 66.7% (2) | 33.3% (1) | 66.7% (2) | 0.00% (0) | 66.7% (2) | 0.00% (0) |
| *S. marcescens* | 1.47% (1) | 100% (1) | 0.00% (0) | 100% (1) | 0.00% (0) | 0.00% (0) | 0.00% (0) | 0.00% (0) |
| *K. oxytoca* | 1.47% (1) | 0.00% (0) | 100% (1) | 0.00% (0) | 100% (1) | 0.00% (0) | 0.00% (0) | 0.00% (0) |
| *K. aerogenes* | 1.47% (1) | 100% (1) | 0.00% (0) | 100% (1) | 0.00% (0) | 0.00% (0) | 0.00% (0) | 0.00% (0) |
| *C. freundii* | 1.47% (1) | 0.00% (0) | 100% (1) | 0.00% (0) | 100% (1) | 0.00% (0) | 0.00% (0) | 100% (1) |
| *Enterobacterales* (subtotal) | 85.3% (58) | 43.1% (25) | 56.9% (33) | 43.1% (25) | 58.6% (34) | 0.00% (0) | 39.7% (23) | 5.17% (3) |
| *P. putida* | 4.41% (3) | 0.00% (0) | 100% (3) | 0.00% (0) | 0.00% (0) | 100% (3) | 0.00% (0) | 0.00% (0) |
| *A. lwoffii* | 2.94% (2) | 0.00% (0) | 100% (2) | 0.00% (0) | 100% (2) | 0.00% (0) | 0.00% (0) | 0.00% (0) |
| *P. alcaligenes* | 2.94% (2) | 0.00% (0) | 100% (2) | 0.00% (0) | 0.00% (0) | 100% (2) | 0.00% (0) | 0.00% (0) |
| *S. maltophilia* | 1.47% (1) | 0.00% (0) | 100% (1) | 0.00% (0) | 0.00% (0) | 0.00% (0) | 0.00% (0) | 0.00% (0) |
| *S. paucimobilis* | 1.47% (1) | 0.00% (0) | 100% (1) | 0.00% (0) | 0.00% (0) | 0.00% (0) | 0.00% (0) | 0.00% (0) |
| *C. indologenes* | 1.47% (1) | 0.00% (0) | 100% (1) | 0.00% (0) | 0.00% (0) | 0.00% (0) | 0.00% (0) | 0.00% (0) |
| Non-fermentative bacilli (subtotal) | 14.7% (10) | 0.00% (0) | 100% (10) | 0.00% (0) | 20.0% (2) | 50.0% (5) | 0.00% (0) | 0.00% (0) |
| Total | 68 | 36.8% (25) | 63.2% (43) | 36.8% (25) | 53.9% (36) | 7.35% (5) | 33.8% (23) | 4.41% (3) |

No blaIMP or blaOXA-48-like were detected. #One isolate was phenotypically identified as KPC but carried blaKPC and blaNDM. CBP: carbapenemase; ESBL: extended-spectrum betalactamase; KPC: Klebsiella pneumoniae carbapenamase; MBL: metallo-beta-lactamase.

**Table S2.** Distribution of CPB isolates and phenotypic carbapenemases in samples (n=1120) used to validate the mBCT against culture (by NA).

| Species | % of isolates (n) | KPC [% (n)] | MBL [% (n)] | Oxa-48-like [% (n)] |
| --- | --- | --- | --- | --- |
| *K. pneumoniae^#^* | 87.0% (94) | 63.8% (60) | 35.1% (33) | 2.13% (2) |
| *E. cloacae* | 5.56% (6) | 83.3% (5) | 16.7% (1) | 0,00% (0) |
| *E. coli* | 2.78% (3) | 33.3% (1) | 66.7% (2) | 0,00% (0) |
| *S. marcescens^#^* | 1.85% (2) | 100% (2) | 50% (1) | 0,00% (0) |
| *K. oxytoca* | 0.93% (1) | 0,00% (0) | 100% (1) | 0,00% (0) |
| *K. aerogenes* | 0.93% (1) | 100% (1) | 0,00% (0) | 0,00% (0) |
| *P. mirabilis* | 0.93% (1) | 0,00% (0) | 100% (1) | 0,00% (0) |
| Total | 108 | 63.9% (69) | 36.1% (39) | 1.85% (2) |

*^#^*In both cases, 1 isolate co-expressed KPC and MBL. KPC: *Klebsiella pneumoniae* carbapenamase; MBL: metallo-beta-lactamase

**Table S3.** Distribution of CPB isolates and phenotypic carbapenemases in samples (n=523) used to compare the Chrom-NA against the CLED-NA.

| Species | CLED-NA | | | Chrom-NA | | | Total [% (n)] |
| --- | --- | --- | --- | --- | --- | --- | --- |
|  | % of isolates (n) | KPC [% (n)] | MBL [% (n)] | % of isolates (n) | KPC [% (n)] | MBL [% (n)] |  |
| *K. pneumoniae^#^* | 86.0% (37) | 64.9% (24) | 37.8% (14) | 86.0% (49) | 61.2% (30) | 40.8% (20) | 86,0% (86) |
| *K. aerogenes* | 0.00% (0) | - | - | 1.75% (1) | 100% (1) | 0.00% (0) | 1,00% (1) |
| *E. coli* | 4.65% (2) | 50.0% (1) | 50.0% (1) | 5.26% (3) | 33.3% (1) | 66.7% (2) | 5,00% (5) |
| *E. cloacae* | 6.98% (3) | 66.7% (2) | 33.3% (1) | 5.26% (3) | 66.7% (2) | 33.3% (1) | 6,00% (6) |
| *P. mirabilis* | 2.33% (1) | 0.00% (0) | 100% (1) | 1.75% (1) | 0.00% (0) | 100% (1) | 2,00% (2) |
| Subtotal | 43 | 62.8% (27) | 39.5% (17) | 57 | 59.6% (34) | 42.1% (24) | 100 |

^#^From both CLED-NA and Chrom-NA, one isolate co-expressed KPC and MBL, corresponding to the same patient. Carriage concordance: 76.4% (n=42). Isolate concordance: 70.9% (n=39). KPC: *Klebsiella pneumoniae* carbapenamase; MBL: metallo-beta-lactamase.

**Table S4.** Main results from the ARIMA model.

| Parameter | Coefficient | Std. Error | 95% CI (Lower–Upper) | p-value |
| --- | --- | --- | --- | --- |
| Intercept | 0.1395 | 0.021 | 0.098 – 0.181 | <0.001 |
| Intervention | -0.059 | 0.03 | −0.118 – 0.000 | 0.051 |
| AR(1) (lag 1) | -0.2832 | 0.646 | −1.549 – 0.983 | 0.661 |
| Residual variance (σ²) | 0.0033 | 0.001 | 0.001 – 0.005 | 0.003 |

Log-likelihood: 23.0; AIC: -38.1; BIC: -35.0; Ljung–Box test (Q): 0.1; Ljung–Box p-value: 0.75; Jarque–Bera p-value: 0.2.

**Figure S1.** Interrupted time-series analysis of CPB prevalence using an ARIMA(1,0,0) model.


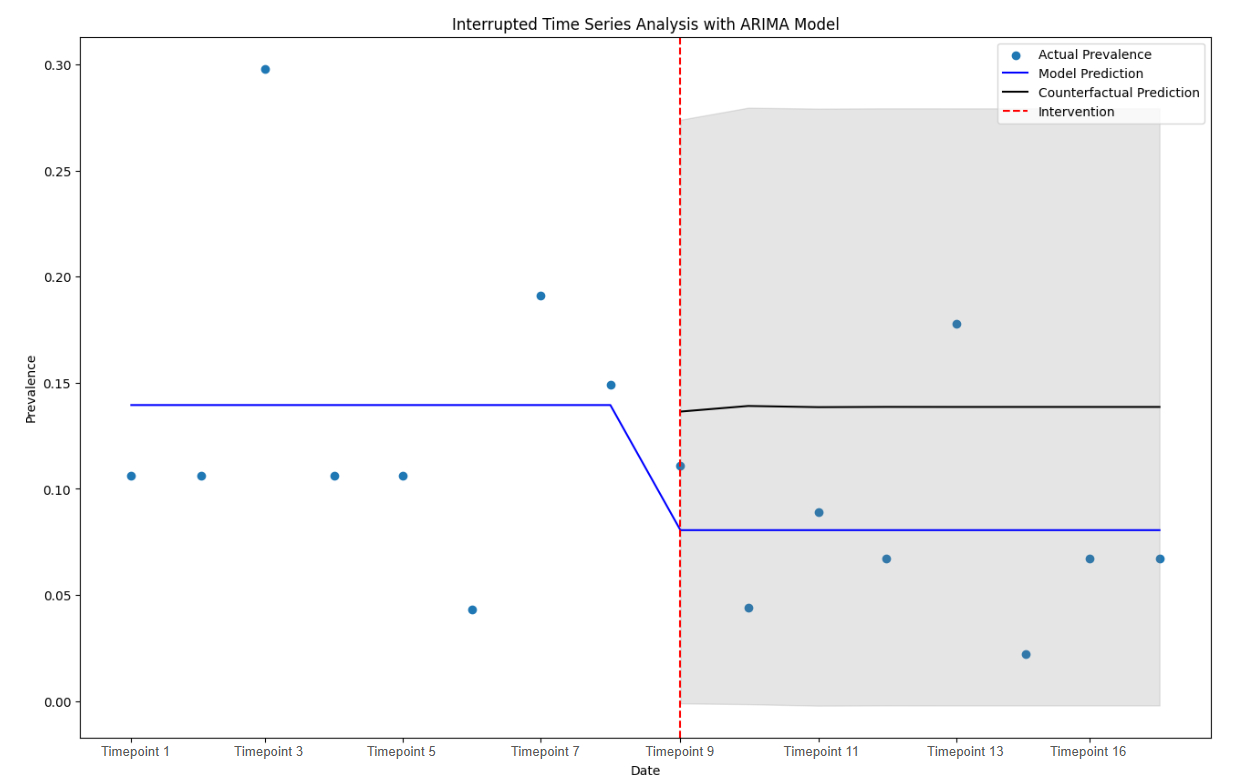


Dots represent observed monthly prevalence. The vertical dashed red line indicates the start of the intervention. The blue line shows the model's predicted prevalence including the intervention effect, while the black line represents the counterfactual forecast had the intervention not occurred. The shaded area shows the 95% confidence interval of the counterfactual.

**Figure S2.** Additive decomposition of the monthly CPB prevalence time series.

**
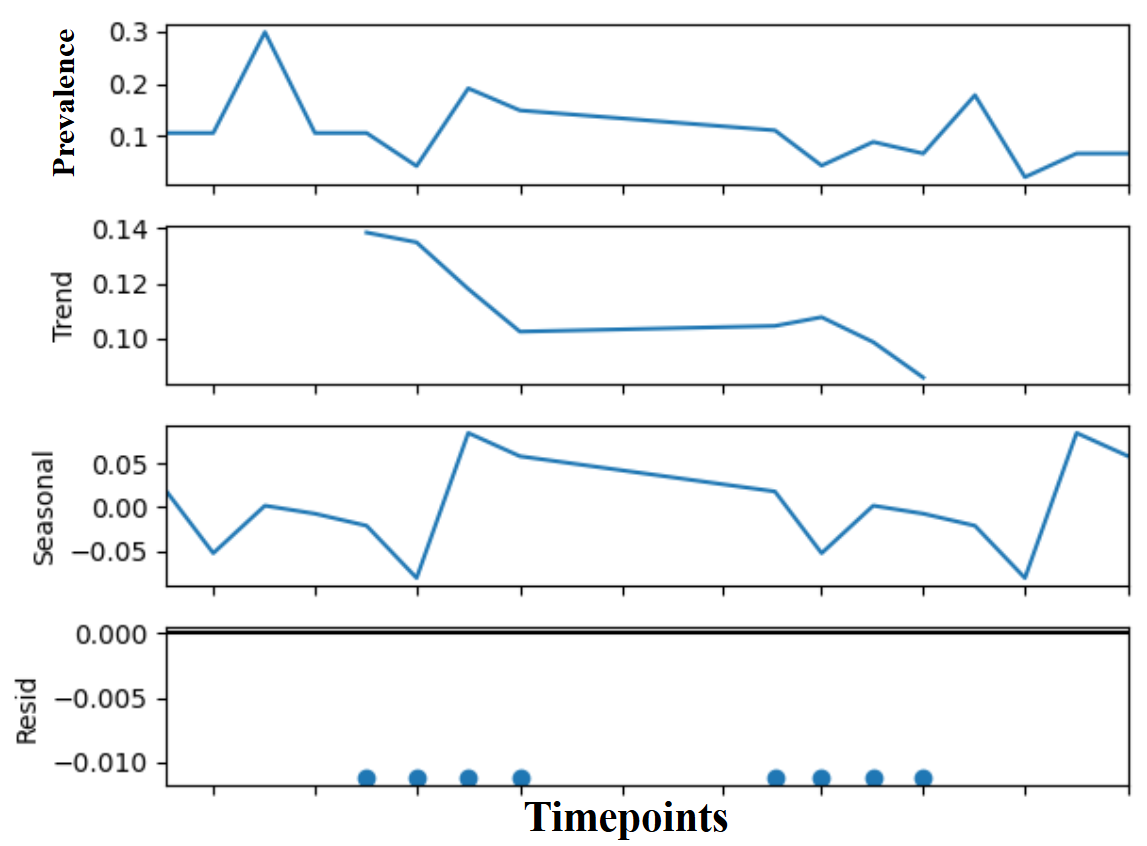
**

The top panel shows the observed prevalence, followed by estimated trend, seasonal component, and residuals.
